# Id1 and Id3 expression is associated with increasing grade of prostate cancer: Id3 preferentially regulates CDKN1B

**DOI:** 10.1002/cam4.19

**Published:** 2012-08-28

**Authors:** Pankaj Sharma, Divya Patel, Jaideep Chaudhary

**Affiliations:** Department of Biological Sciences, Centre for Cancer Research and Therapeutics Development, Clark Atlanta UniversityAtlanta, Georgia, 30314

**Keywords:** Id1, Id2, Id3, inhibitor of differentiation, prostate cancer

## Abstract

As transcriptional regulators of basic helix–oop–helix (bHLH) transcription and non-bHLH factors, the inhibitor of differentiation (Id1, Id2, Id3, and Id4) proteins play a critical role in coordinated regulation of cell growth, differentiation, tumorigenesis, and angiogenesis. Id1 regulates prostate cancer (PCa) cell proliferation, apoptosis, and androgen independence, but its clinical significance in PCa remains controversial. Moreover, there is lack of evidence on the expression of Id2 and Id3 in PCa progression. In this study we investigated the expression of Id2 and Id3 and reevaluated the expression of Id1 in PCa. We show that increased Id1 and Id3 protein expression is strongly associated with increasing grade of PCa. At the molecular level, we report that silencing either Id1 or Id3 attenuates cell cycle. Although structurally and mechanistically similar, our results show that both these proteins are noncompensatory at least in PCa progression. Moreover, through gene silencing approaches we show that Id1 and Id3 primarily attenuates CDKN1A (p21) and CDKN1B (p27), respectively. We also demonstrate that silencing Id3 alone significantly attenuates proliferation of PCa cells as compared with Id1. We propose that increased Id1 and Id3 expression attenuates all three cyclin-dependent kinase inhibitors (CDKN2B, -1A, and -1B) resulting in a more aggressive PCa phenotype.

## Introduction

The inhibitor of DNA-binding (Id) proteins, Id1–4, are negative regulators of basic helix–loop–helix (bHLH) transcription factors. The repertoire of Id-regulated cellular pathways is large and diverse due to their ability to interact and modulate the activity of bHLH and non-bHLH transcription factors and regulatory molecules (reviewed in [1–5]). As key regulators of cell cycle and differentiation, the expression of Id proteins is increasingly observed in many cancers and in most cases associated with aggressiveness of the disease including poor prognosis [6–9], metastasis [10], and angiogenesis [11, 12]. Of all the four Id proteins, the expression of Id1 and Id2 in cancer and the underlying molecular mechanism is relatively well known. Recent investigations also support the role of Id3 in cancer. On the contrary, epigenetic silencing of Id4 in many cancers tends to support its role as a tumor suppressor [13].

In spite of strong evidence supporting the role Id1 as a tumor promoter, its expression in prostate cancer is conflicting [9, 14–19]. Majority of studies have shown that Id1 protein expression is increased with increasing grade of prostate cancer [9, 14, 15–18] that is associated with decreased apoptosis, increased proliferation and metastasis, androgen independence, and altered signaling pathways, such as epidermal growth factor receptor (EGFR) (reviewed in [9, 14–18]). However, a recent study using a highly specific human Id1 rabbit monoclonal antibody showed no association with protein expression in prostate cancer [19]. These results prompted us to re-evaluate the association between Id1 and prostate cancer using the same rabbit monoclonal antibody [19].

Observations have suggested heterogeneity in Id1 and Id3 possibly due to high degree of sequence similarity: both Id1 and Id3 are required for neurogenesis, angiogenesis, and vascularization of tumor xenografts [11] and involved in breast cancer lung metastasis [20]. Recent results, however, suggest that Id1 may target unique pathways that are distinct from Id3: Id1 but not Id3 appears to direct long-term repopulating hematopoietic stem cell maintenance [21]. Surprisingly, Id3^−/−^ mouse were shown to develop *γδ* T-cell lymphoma [22], suggesting a tumor suppressive role, at least in hematological malignancies. In gastric cancer, Id3, but not Id1, was a strong independent predictor for shorter overall survival [7]. Although we demonstrated that Id3 is expressed in prostate cancer cell lines, its expression in prostate tissue was not investigated [23].

The purpose of this study was to investigate the expression and relevance of Id1 and Id3 proteins in prostate cancer. The results demonstrate that Id1 and Id3 expression is associated with prostate cancer. We also demonstrate that Id3 alone blocked proliferation of prostate cancer cells as compared with Id1. Although both Id1 and Id3 independently regulate CDKNI-dependent cell cycle, Id3 appears to regulate CDKN1B (p27), whereas Id1 primarily regulates CDKN1A (p21). Our results suggest that increased Id1/Id3 could lead to downregulation of all three CDKNIs resulting in aggressive phenotype in prostate cancer.

## Materials and Methods

### Cell culture and Id silencing

Human prostate cancer cell lines LNCaP, DU145, and PC3 were obtained from American Type Culture Collection (ATCC, Rockville, MD) and cultured as reported previously [23] in 5% fetal bovine serum (FBS [PAA Labs, New Bedford, MA]). Id1 and Id3 were transiently silenced by gene specific siRNA as previously described [23, 24] in the presence of serum (5% FBS) unless noted otherwise.

### Western blot analysis

Cells were lysed using mammalian protein extraction reagent (Pierce, Rockford, IL) with protease inhibitors (complete mini, Roche, Indianapolis, IN). Forty microgram of protein was electrophoretically separated on 12% sodium dodecyl sulfate-polyacrylamide gel electrophoresis and blotted onto nitrocellulose membranes (Millipore, Billerica, MA). Western blotting was performed according to standard procedures. After incubation with primary (Biocheck - Id1: 195-14 [1:2000 dilution] and Id3: 6-1 [1:2000], Santa Cruz – p27: sc776 [1:3000], p21:sc-471 [1:1000], p16: sc-468 [1:2000]) and secondary antibodies (SA1-9510, horseradish peroxidase (HRP)-goat anti-rabbit [1:5000], Thermo Scientific, Rockford, IL), the membranes were developed using enhanced chemiluminescence (GE Healthcare Life Sciences, Piscataway, NJ) and blots visualized and semiquantitated using the Fuji Film LAS-3000 Imager.

### Immunohistochemistry (IHC) of tissue microarray slides

Prostate cancer tissue microarrays were used to investigate Id1 and Id3 expression. In all, Id1 and Id3 expression was analyzed in 41 prostate cancers (mean age 70 ± 7.9, grade I: *n* = 9, grade II: *n* = 14, grade III: *n* = 18), six benign prostatic hyperplasia (BPH) (mean age 73 ± 4.6), and eight normal (mean age 53.35 ± 16.5) prostate core biopsies (1.5 mm) in duplicate (BC19014, BC19111, and T192, US BioMax, Inc., Rockville, MD). The cancer grade and histological type information were available from the manufacturer for each of the sections. The prostate cancer grading (as provided by the manufacturer US BioMax) was as follows: grade I, well differentiated; grade II, moderately differentiated; grade III, poorly differentiated.

Tissue microarray slides were deparaffinized in xylene and rehydrated through standard protocols. Antigens were retrieved by autoclaving in 0.01 mol/L sodium citrate buffer pH 6.0 at 121°C/20 psi for 30 min. The peroxidase activity was blocked in 3% H_2_O_2_ and nonspecific binding sites blocked in 10% Goat serum. The blocked sections were incubated overnight at 4°C with primary antibody (1% bovine serum albumin [BSA] in phosphate buffer saline with tween 20 [PBST]) followed by incubation with secondary antibody (SA1-9510, HRP-goat anti-rabbit, Thermo Scientific) for 1 h. The slides were stained with diaminobenzidine for 2 min, counterstained with hematoxylin and mounted with Immuno-mount (Thermo Scientific), examined and photomicrographs taken using the Zeiss fluorescent microscope with an AxoimCam version 4.5 imaging system.

### Semiquantitation of Id expression prostate tissue microarray

The intensity of staining was rated from 0 for below the level of detection to 3 for strongest expression by two independent observers to determine the change in Id expression during prostate cancer progression. The observers were only informed about the antibody being scored. The correlation coefficient between the assessment of Id staining by two independent observers was *r* = 0.93–0.96.

### Immunocytochemistry

Cells were grown on glass chamber slides up to 75% confluency. The slides were then washed with phosphate buffer saline (PBS) [3] and fixed in ice-cold methanol for 10 min at room temperature and stored at −20°C until further use. Before use, the slides were equilibrated at room temperature and washed with PBS (5 min, 3×). Cells were then blocked with 1% BSA in PBST for 30 min at room temperature and incubated with primary antibody (1% BSA in PBST) for overnight at 4°C, washed in PBS, and incubated with secondary antibody with fluorochrome (Goat anti-rabbit-IgG [H+L] DyLight 594 [red] or 488 [green] conjugated, Thermo Scientific) in 1% BSA for 1 h at room temperature in dark. The slides were subsequently washed again and stained in 4′,6-diamidino-2-phenylindole (1 *μ*g/mL) for 1 min and mounted with glycerol. Images were acquired by Zeiss fluorescence microscope through Axiovision software.

### Cell proliferation and cell cycle analysis following Id1 and Id3 silencing

The proliferation rate as reflected by rate of DNA synthesis was performed using ^3^H thymidine incorporation assays as previously described [25]. Cell cycle distribution was determined by staining DNA with propidium iodide (PI) (Calbiochem, Billerica, MA). Briefly, transiently Id1, Id3, or Id1+Id3 silenced LNCaP and DU145 cells were harvested, washed in ice-cold PBS, and fixed in 70% ethanol. Cell pellets were suspended with PI with simultaneous RNase treatment at 37°C for 30 min in dark. The number of cells in the different phases of the cell cycle were measured with Accuri C6 flow cytometer and data were analyzed using CFlow Plus software (Accuri Cytometers, Inc., San Jose, CA)

### Quantitative PCR analysis

The relative gene expression levels of selected genes were determined by real-time quantitative polymerase chain reaction (PCR) based on TaqMan chemistry using Applied Biosystems probes (TaqMan Probes, Applied Biosystems, Foster city, CA). All PCR reactions were performed in a final volume of 50 *µ*L. The cycle threshold (Ct) was used to calculate relative amounts of target RNA. All experiments were performed in duplicates and repeated thrice. The ΔΔCt method was used for relative quantification of gene expression as previously described [24].

### Statistical analysis

Semiquantitative analysis of Id expression in normal prostate, BPH, and prostate cancer (grades I–III) was evaluated by nonparametric Kruskal–Wallis statistics (nonparametric one-way analysis of variance [ANOVA]) followed by post hoc Dunn's test (GraphPad Prism v5). Student's *t*-test was used for all other comparisons.

## Results

### Id1 and Id3 expression in the prostate

**Figure 1:**
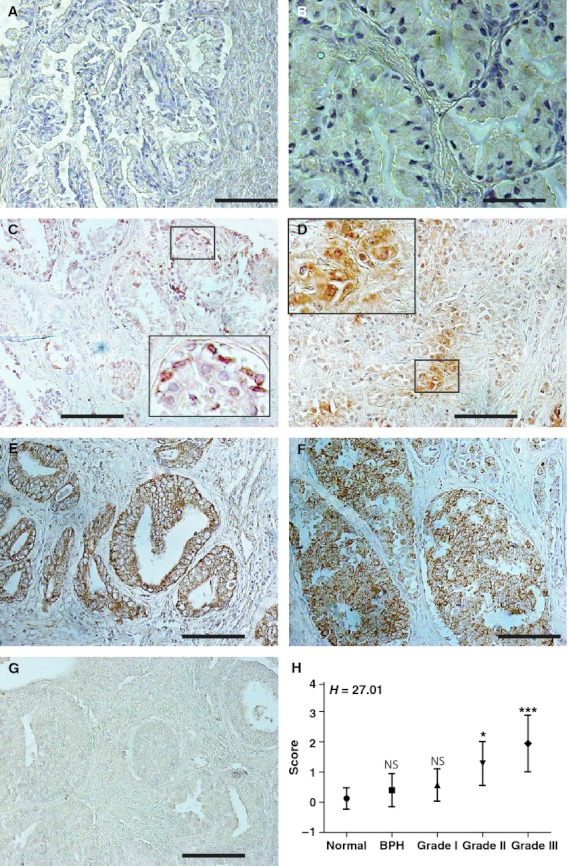
Prostate cancer tissue microarrays were used to investigate Id1 expression. (G) represents the negative control. Id1 is low to absent in normal prostate (A: 200× and B: 400×). Id1 expression increases with increasing grade of prostate cancer (C: grade I [200×, inset is the enlarged boxed region], D: grade II [200×, inset is the enlarged boxed region], E: grade III [200×], and F: 400×, respectively). (G) Grade III cancer section stained with Id1 antibody in the presence of recombinant human Id1. The brown staining is indicative of Id1 expression and blue staining represents nuclei. Representative images are shown. (H) Semiquantitative analysis of Id1 expression in normal prostate, BPH, and prostate cancer (grades I–III). The Kruskal–Wallis statistics *H* was 27.01 indicating significant differences between groups. The post hoc Dunn's test was used to determine statistical differences between groups: NS, nonspecific; *P < 0.05 and ***P < 0.001. Data are represented as mean ± standard deviation (SD). Id1 staining intensity was scored as follows: Panels A and B were scored as 0, C and D were scored as 1, and E and F were scored as 3. The scale bars are 200 *µ*m.

Id1 expression was essentially undetectable in normal prostate epithelial cells (Fig. 1A and B). Id1 expression was also not observed in the stromal compartment in either normal prostate (Fig. 1A and B) or prostate cancer (Fig. 1C–F). In contrast, high Id1 expression was observed in majority of prostate adenocarcinoma (Fig. 1C–F) and was localized primarily to the glandular epithelial cells (Fig. 1C and D and inset). Out of observed cases of 18 grade III prostatic adenocarcinoma, 17 specimens showed strong to moderate Id1 expression and one with no expression. In Id1 positive prostate (grades I through III) cancer specimens, Id1 staining was nuclear (Fig. 1C and D and inset), but cytoplasmic staining was also observed (Fig. 1E and F, grade III, 200× and 400×, respectively).

**Figure 2:**
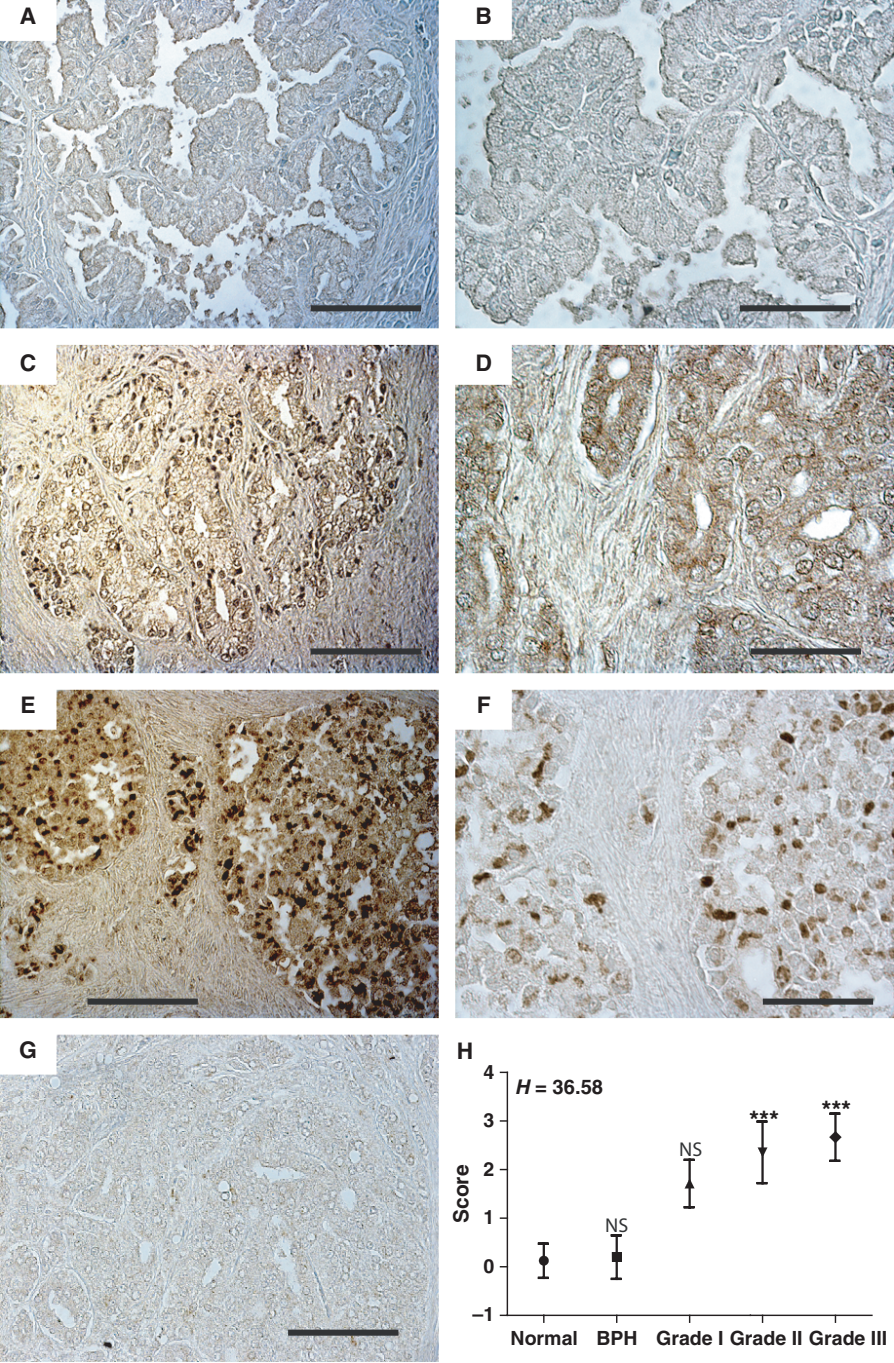
Prostate cancer tissue microarrays were used to investigate Id3 expression. Id3 (brown staining) was low to absent in normal prostate (A: 200× and B: 400×). Id3 expression increases with increasing grade of prostate cancer (C: grade II, E and G: grade III [200× and 400×, respectively]). (G) represents negative control. (D) 400× image of grade III cancer showing cytoplasmic/perinuclear Id3 expression as compared with nuclear expression in panels E and F. Representative images are shown. (G) Grade III cancer section stained with Id3 antibody in the presence of recombinant human Id3. (H) The Kruskal–Wallis statistics *H* was 36.58 indicating significant differences between groups. The post hoc Dunn's test was used to determine statistical differences between groups: NS, nonspecific; ***P < 0.001. Data are represented as mean ± standard deviation (SD). Id3 staining intensity was scored as follows: Panels A and B were scored as 0, C and D were scored as 2, and E and F were scored as 3. The scale bars are 200 *µ*m.

**Figure 3:**
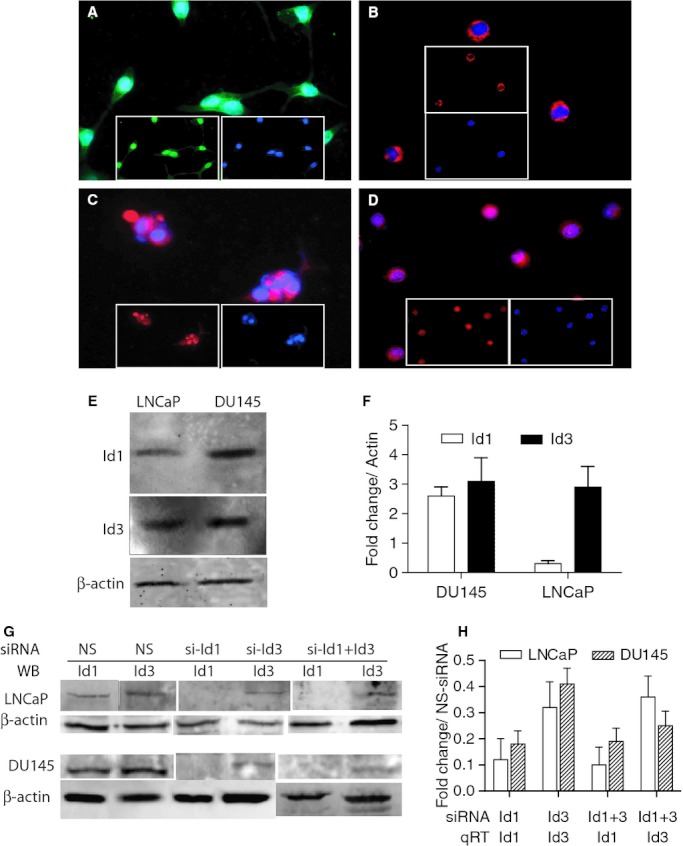
(A–D) Expression and cellular localization of Id1 (A and B) and Id3 (C and D) in prostate cancer cell lines LNCaP (A and C) and DU145 (B and D) at 400× magnification by fluorescent immunocytochemistry. The fluorescent images are composite merged images (inset) of Id1 (green, A: LNCaP; and red, B: DU145), Id3 (red, C and D), and 4′,6-diamidino-2-phenylindole (blue, nuclear, inset). The representative of at least four different experiments is shown. (E) Western blot analysis of Id1 and Id3 expression in LNCaP and DU145 cells. (F) Semiquantitative analysis (densitometry) of Id1 and Id3 expression in LNCaP and DU145 prostate cancer cell lines by Western blot analysis. The data normalized to actin are represented as mean ± SEM of at least three different experiments. (G) Western blot analysis of Id1 and Id3 expression in LNCaP and DU145 cells silenced with corresponding gene specific siRNA. The constitutively expressed *β*-actin was used as loading control. The blot is representative of at least three experiments. WB, Western blot; NS, nonspecific siRNA. (H) Real-time PCR-based quantitative analysis of Id1 and Id3 expression followed by silencing with the corresponding siRNA in LNCaP and DU145 cells. Id1 and Id3 expression was also analyzed in cells silenced with combined Id1 and Id3 siRNA. The data (mean ± SEM) represent fold change as compared to cells transfected with corresponding nonspecific siRNA in LNCaP and DU145 cells.

Id3 was essentially undetectable or expressed at low levels in the normal prostate tissue (Fig. 2A and B). A very significant increase in Id3 expression was observed in prostate cancer (grade II, Fig. 2C). Overall, a positive correlation between Id3 expression and prostate cancer grade was observed (Fig. 2C and E). In grade I and II prostate cancer specimens, Id3 expression was predominantly cytoplasmic to perinuclear, but in some cells intense nuclear Id3 expression was also observed (Fig. 2C and D). The intensity of Id3 staining in the nucleus increased dramatically in grade III cancers, although a weak cytoplasmic staining still persisted, a pattern which is similar to that observed in prostate cancer cell lines (Fig. 3C and D). We speculate that Id3 undergoes a distinct cytoplasmic-nuclear shuttling with increasing grades of prostate cancer, although the significance of this shuttling remains unclear.

Lack of Id1 and Id3 immunoreactivity on tissue microarray slides using respective Id1 and Id3 recombinant proteins (Figs. 1E and 2G) demonstrated specificity of the antibodies used in this study. No crossreactivity between Id1 antibody and Id3 recombinant protein and vice versa was observed in enzyme-linked immunosorbent assay (ELISA) (data not shown).

Semiquantitative analysis of Id1 and Id3 expression in prostate cancer specimens using nonparametric Kruskal–Wallis analysis essentially validated our observations stated above: increased Id1 (Fig. 1H) and Id3 (Fig. 2H) expression was significantly associated with increasing grade of prostate cancer. Interestingly a stronger statistical association was observed in case of Id3 with prostate cancer as compared with Id1: the difference between Id3 expression in normal versus grade II was more significant in post hoc Dunn's multiple comparison test (***P < 0.001, Fig. 2H) as compared with Id1 (*P < 0.05, Fig. 1H).

### Id1 and Id3 expression and significance in prostate cancer cell lines LNCaP and DU145

#### Id1 and Id3 expression in prostate cancer cell lines

Detailed cellular localization of Id1 and Id3 proteins in prostate cancer cell lines was investigated by immunocytochemistry (Fig. 3A–D, Id1 and Id3 expression shown in LNCaP and DU145 cells). Id1 (Fig. 3A) and Id3 (Fig. 3C and D) demonstrated both nuclear and cytoplasmic localization in LNCaP (Fig. 3A and C) and DU145 (Fig. 3B and D) cells, respectively. The localization of Id1 and Id3 is also consistent with their localization in prostate cancer tissue.

The prostate cancer cell lines LNCaP and DU145 expressed Id1 and Id3 as determined by Western blot analysis (Fig. 3E, Id1 and Id3 in LNCaP and DU145). Semiquantitative analysis of Western blot indicated that Id3 is constitutively expressed at significantly higher levels as compared with Id1 in cell lines (Fig. 3F), but Id1 expression was more dynamic between cell lines with a following expression pattern: DU145 >> LNCaP.

#### Id1 and Id3 silencing attenuates proliferation

**Figure 4:**
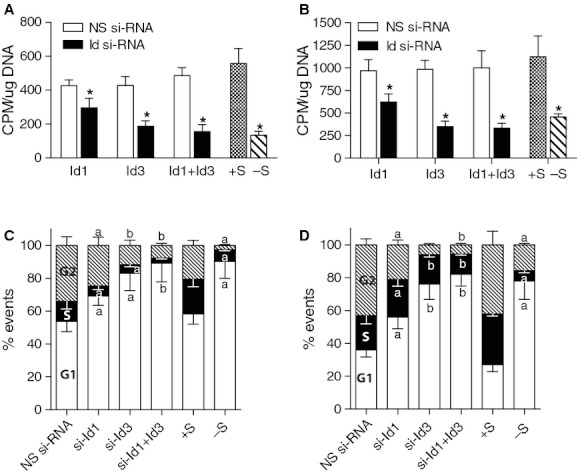
Effect of Id1 and Id3 silencing on cell proliferation and cell cycle. ^3^H-thymidine-based analysis of DNA synthesis in LNCaP (A) and DU145 (B) cells silenced with either Id1, Id3, or a combination of Id1 and Id3 was used to measure rate of proliferation. The data are expressed as counts per minute (CPM) normalized to total DNA (*P < 0.001, significant differences between cells transfected with NS or gene specific siRNA or between +S and −S). The data are expressed as mean + SEM of three experiments. The cell proliferation in the presence of serum (+S) and absence of serum (serum starved for 48 h: −S) was used to assess the rate of maximum and minimum proliferation, respectively, in both cell lines. The cells transfected with either nonspecific siRNA (NS) or gene specific siRNA as indicated above was also used to quantitate cells in different phases of cell cycle (C: LNCaP and D: DU145). (a) Significant difference between NS and siRNA samples or between +S and −S, (b) significant difference between cells transfected with gene specific siRNA (e.g., between si-Id1 and si-Id3).

**Figure 5:**
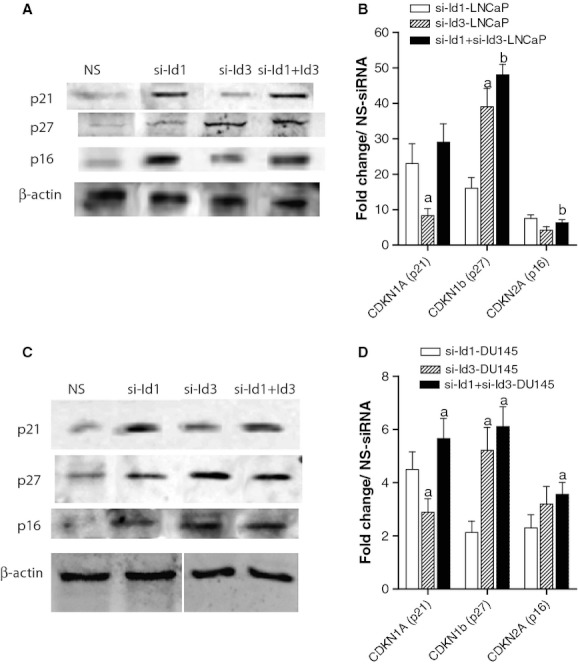
Effect of silencing Id1, Id3, or Id1 + Id3 on the expression of cyclin-dependent kinase inhibitors CDKN1B (p27), CDKN1A (p21), and CDKN2B (p16). Panels A and C are the Western blot–based protein expression of all three CDKNIs in LNCaP (A) and DU145 (C) cells. *β*-Actin was used as loading control. The blot shown is representative of three different experiments. Panels B and D are the real-time PCR-based quantitative expression of CDKNIs in LNCaP (C) and DU145 (D) cells silenced with either Id1, Id3, or Id1 + Id3. Data (mean ± SEM) are expressed as fold change in the expression of CDKNIs in the presence of gene specific siRNA as compared with nonspecific RNA. NS, nonspecific siRNA, (a) significant (*P* < 001) as compared with si-Id1, (b) significant as compared with Id3.

Increased proliferation is a well-established hallmark of cancer cells that is known to be regulated by Id1 and Id3 [23, 24]. We have previously reported that silencing either Id1 or Id3 in prostate cancer cell lines LNCaP and DU145 cells independently attenuated proliferation [23]. These results also demonstrated that silencing Id1 had no effect on the expression of Id3 and vice versa, suggesting that Id1 and Id3 could possibly regulate unique cell cycle regulatory mechanisms [24]. We investigated the effect of silencing Id1 and Id3 either alone or in combination (Fig. 3G and H) to dissect possible Id1- or Id3-dependent mechanisms on the proliferation of LNCaP and DU145 cells (Fig. 4). Consistent with our previous observation, silencing either Id1 or Id3 significantly reduced proliferation in LNCaP (Fig. 4A) and DU145 cells (Fig. 5B) [23]. Interestingly, silencing of Id3 alone reduced proliferation to a greater degree than Id1 on both cell lines (Fig. 4A and B). The magnitude of decrease in proliferation in Id3 silenced cells was not due to higher degree of Id3 silencing as compared with Id1, as Id3 expression was reduced only by 32% and 41% in LNCaP and DU145 cell, respectively, as compared with almost undetectable levels of Id1 after siId1 transfection in both LNCAP and DU145 cell lines (Fig. 3 G and H). The effect of silencing Id1 and Id3 together on proliferation of LNCaP and DU145 cells was not additive, but was similar to the levels observed after Id3 silencing alone (Fig. 4A and B). We speculated and confirmed that Id3 silencing alone was enough to induce a complete block in proliferation by comparing the rate of proliferation in serum starved LNCaP and DU145 cells (Fig. 4A and B; +S: with serum and −S: serum starved for 48 h).

#### Loss of Id1 and Id3 promotes G1 arrest

Flow cytometery–based cell cycle analysis demonstrated that Id1 and Id3 silencing promoted a G1 arrest (Fig. 4C and D). The percentages of cells in G1 phase following Id3 (83 ± 10.52%) and Id1+Id3 (89.2 ± 11.3%) were not statistically different suggesting that Id3 blocks cell cycle to a greater degree than Id1 alone (69.2 ± 5.5) in LNCaP cells (Fig. 4C). Similar results were also obtained in DU145 cells (Fig. 4D). These results strongly suggest that the profound effect of Id3 on proliferation could be due to unique molecular mechanisms that are not compensated by Id1.

#### Id1 and Id3 regulate CDKNIs

In order to further investigate the mechanism by which Id1 and Id3 alters proliferation as discussed above, we investigated the effect of silencing Id1, Id3, and Id1+Id3 on cyclin-dependent kinase inhibitors, the key cell cycle regulatory genes that are also direct Id1 and Id3 targets. The real-time quantitative PCR analysis suggested that silencing Id1, Id3, or Id1+Id3 increased the expression of all three CDKNIs, as expected. The transcript and protein expression data, however, pointed toward a distinct Id1- or Id3-dependent regulatory mechanism: silencing Id3 alone significantly increased the expression of p27 in LNCaP (39.4-fold, Fig. 5A and B) and DU145 (5.22-fold, Fig. 5C and D) cells as compared with Id1 alone in LNCaP (16-fold, Fig. 5B) and DU145 (2.1-fold, Fig. 5D) cells. Lack of Id1+Id3 further increased the levels of p27 by 48-fold and 6.11-fold in LNCaP (Fig. 5B) and DU145 (Fig. 5D), respectively, as compared with nonsilencing control, but was not statistically different as compared with Id3 alone, suggesting that both these CDKNIs are under greater regulatory control by Id3 as compared with Id1. In contrast, Id1 preferentially regulated p21 in both LNCaP and Du145 cells (Fig. 5A and B). A significant change in the magnitude of expression of p21 was observed between cells silenced with Id1 alone or Id1+Id3 suggesting that p21 is primarily regulated by Id1. Together with cell cycle data, these results suggest that the decrease in cell proliferation and an increase in G1 arrest following Id1, Id3, or Id1+Id3 silencing could be due to significantly higher levels of cyclin-dependent kinase inhibitors. Moreover, our results provide direct evidence that Id1 and Id3 could preferentially regulated p21 and p27, respectively.

## Discussion

This study provides the first comprehensive analysis of Id1 and Id3 protein expression and localization in human prostate cancer.

Despite significant advances in our understanding of the mechanism of action of Id1 in prostate cancer, such as regulation of p16 [26, 27], EGFR [28], androgen independence, prostate-specific antigen expression [29] and its role in cell proliferation [23] and metastasis [30], the expression Id1 has remained controversial. Data presented in this study, from our previous studies [23, 26], and as reported by others [9, 14–18] strongly suggest that Id1 is associated with prostate cancer. A report by Perk et al. [19] demonstrated no expression of Id1 in prostate cancer tumor cells, however, the basal cells, on average, 40% of benign seeming glands were found to express Id1. Such expression was absent in the glands of normal and hyperplastic prostates [19]. The authors linked Id1 expression in basal cells as part of the stem cell compartment [19]. In our studies, we observed a clear Id1 expression in tumor-derived cells. The discrepancy in Id1 staining pattern between our study and that reported by Perk et al. [19] is very significant especially because we used the same antibody that was extensively validated. The reason for this discrepancy is difficult to ascertain but we speculate differences in methodology. Multiple factors collectively could have led to different results: fixation followed by antigen retrieval, dilution, and length of incubation of primary antibody. The previous study [19] also did not specify the antigen retrieval system which could have been different from the one used in this study. The antigen retrieval can significantly alter the binding of the antibody to its epitope especially when using monoclonal antibody as used in these studies. Differences between Id1 expression at the transcript and protein level are also observed. The Id1 transcript as determined by a combination of complementary DNA (cDNA)-based membrane array [31], RT-PCR on RNA isolated from formalin-fixed paraffin-embedded tissue [32], and microarray [33] suggests that Id1 expression is in fact decreased in prostate cancer: these results are clearly contradictory to the majority of protein expression studies as discussed above. A recent study by Yu et al. [17] demonstrated that Id1 mRNA measured by quantitative RT-PCR on RNA prepared from snap frozen tissue and the corresponding protein is also increased in prostate cancer as compared with BPH. It is possible that Id1 transcript is particularly sensitive in terms of how the sample is prepared, for example, the study demonstrating decreased Id1 in a membrane array [31] also showed decreased expression of monocyte chemotactic protein-1 (MCP-1) in prostate cancer. However, subsequent studies demonstrated that MCP-1 expression is increased in prostate cancer [34].

From a functional perspective, Id1 is a transcriptional regulator and not a transcription factor. Moreover, Id1 lacks a nuclear translocation signal, hence it is not unlikely to observe high Id1 expression in the cytoplasm. Cytoplasmic Id1 staining had been reported in a number of studies including prostate [17], breast [35], and gastric [36] cancers. In fact a study by Maw et al. [35] demonstrated diffuse cytoplasmic staining in most cases, whereas nuclear staining was observed only occasionally, results that are similar to those observed in prostate cancer study published elsewhere [17] and in our study. In gastric cancer, Id1 was nuclear in well-differentiated carcinoma, but was cytoplasmic in moderately to poorly differentiated carcinoma [36]. The relevance of cytoplasmic Id1 expression remains unknown, but we speculate that it is involved in multiple interactions with cytoplasmic proteins, such as caveolin [37] and E2A [38] to modulate half-life and/or cellular localization. Based on our and majority of studies, we can now confidently state that increased Id1 is associated with prostate cancer.

The increased expression of Id3 in prostate cancer is a novel observation. Together, increased Id1 and Id3 expression is observed in many cancers and is associated with poor prognosis [7]. At the mechanistic level, Id1 and Id3 are compensatory at least in the knockout mouse model [11]. However, recent reports suggest that Id1 and Id3 could have distinct pathways, for example, Id1 but not Id3 directs long-term repopulating hematopoietic stem-cell maintenance [21]. Our results show that targeting Id3 alone can reduce prostate cancer cell proliferation significantly more as compared with silencing Id1 alone. Silencing Id3 alone in small cell lung carcinoma can also reduce proliferation in spite of persistent Id1 expression [39]. These results clearly demonstrate that Id1 cannot completely restore Id3-dependent cell cycle pathways. The decrease in proliferation in cells lacking both Id1 and Id3 is also not significantly different from cells lacking Id3 alone, further suggesting a dominant role of Id3 in prostate cancer cell proliferation. While both Id1 and Id3 downregulate all three cyclin-dependent kinase inhibitors CDKN1B (p27), CDKN1A (p21), and CDKN2B (p16) leading to increased proliferation, but the mechanism by which specific Id isoform regulates CDKNI expression appears to be different and not necessarily compensatory. A comprehensive and detailed expression of CDKNIs following silencing of Id1, Id3, or both suggested that Id1 preferentially regulates p21 expression as shown previously [40], whereas Id3 is more likely involved in regulating the expression of p27. Collectively, these results support the G1 arrest observed following either Id1 or Id3 silencing in LNCaP and DU145 cells. In mice, p27 is a tumor suppressor and its loss is a negative prognostic indicator in many cancers. In a functional genomic screen, Id3 was identified as a transcriptional repressor of Id3 [41]. A study by Chassot et al. [42] demonstrated that Id3 is involved in transcriptional repression CDKN1B (p27) in human dermal fibroblasts. Subsequent reporter gene experiments and chromatin immunoprecipitation assay demonstrated that Id3 likely exerts its repressive action on p27 transcription through ELK1 (an ETS family transcription factor) inhibition [42]. Although we did not investigate the detailed mechanism at the promoter level in this study, but it is speculated that the Id1- and Id3-dependent mechanism could involve a combination of both bHLH-dependent and -independent mechanisms.

Studies have shown that decreased p16 and p27 expression is associated with prostate cancer. Silencing both p21 and p27 but not individually in a DU145 cell line–based xenograft model produces a more aggressive prostate cancer phenotype with increased angiogenesis [43, 44]. Low p16 levels are also associated with higher risk of distant metastasis [45]. Our studies have also shown that ectopic Id1 expression alone promotes p16-dependent immortalization of prostate epithelial cells [26]. Increased Id1 and Id3 expression could therefore significantly decrease the expression of CDKNIs as shown in this and other studies that could be a mechanism leading to aggressive phenotype in prostate cancer. In spite of strong sequence similarity and assumed functional redundancy, the function of Id3 in promoting a cancer phenotype now appears to be distinct from Id1. The parallel impact of suppressed Id1 and 3 on these two very different cell lines implies a core role for the Id proteins in regulation of G1/S transition in prostate cancer cells that is independent of known differences between the two cell lines, for example, androgen receptor status, phosphatase and tensin homolog status, p53 and Rb mutations.

In conclusion, our results clearly demonstrate that Id1 and Id3 expression is associated with prostate cancer progression. A number of studies had shown that Id1 is a potential therapeutic target in prostate cancer. Our results suggest that Id3 could be a more potent therapeutic target than Id1 based on our gene silencing and corresponding proliferation/cell cycle and CDKNI expression studies. We propose that Id1 and Id3 together could have higher diagnostic and therapeutic value in prostate cancer. A similar peptide aptamer–based approach targeting both Id1 and Id3 (Id1/3-PA7) was shown to induce cell cycle arrest and apoptosis in breast cancer cells MCF7 and MDA-MB-231 [46]. Aptamer or small molecule inhibitor that could target HLH domain of Id1 and Id3 could therefore be an ideal therapeutic approach in prostate cancer.
